# Proof-of-concept and concurrent validity of a prototype headset to assess peak oxygen uptake without a face mask

**DOI:** 10.1186/s13104-021-05850-y

**Published:** 2022-01-08

**Authors:** Peter Düking, Philipp Kunz, Florian A. Engel, Helena Mastek, Billy Sperlich

**Affiliations:** Integrative and Experimental Exercise Science and Training, Department of Sport Science, University of Würzburg, Würzburg, Germany

**Keywords:** Digital Health, eHealth, mHealth, Innovation, Data-informed training, Wearable

## Abstract

**Objective:**

Portable gas exchange instruments allow the assessment of peak oxygen uptake (V̇O_2peak_) but are often bulky, expensive and require wearing a face mask thereby limiting their routine application. A newly developed miniaturized headset (VitaScale, Nuremberg, Germany) may overcome these barriers and allow measuring V̇O_2peak_ without applying a face mask. Here we aimed (i) to disclose the technical setup of a headset incorporating a gas and volume sensor to measure volume flow and expired oxygen concentration and (ii) to assess the concurrent criterion-validity of the headset to measure V̇O_2peak_ in 44 individuals exercising on a stationary cycle ergometer in consideration of the test–retest reliability of the criterion measure.

**Results:**

The coefficient of variation (CV%) while measuring V̇O_2peak_ during incremental cycling with the headset was 6.8%. The CV% for reliability of the criterion measure was 4.0% for V̇O_2peak_. Based on the present data, the headset might offer a new technology for V̇O_2peak_ measurement due to its low-cost and mask-free design.

**Supplementary Information:**

The online version contains supplementary material available at 10.1186/s13104-021-05850-y.

## Introduction

Cardiorespiratory fitness, expressed as peak oxygen uptake (V̇O_2peak_) is a key determinant of mortality in the general population [1] and an important limiting factor of endurance performance [2]. During cardiopulmonary exercise testing, V̇O_2peak_ is commonly determined with portable or stationary gas and volume analyzers with the intention of assessing cardiorespiratory fitness or cardiorespiratory response to different interventions [3, 4]. In contrast to bulky stationary instruments (e.g. Douglas-bag or mixing chamber procedures), portable breath-by-breath gas analyzers allow in-field measurements of an individual´s respiratory cycle with rapidly alternating exercise intensity [5, 6]. However, current portable breath-by-breath instruments are expensive and require specialized staff for analysis and interpretation of data. Also, the determination of V̇O_2peak_ with common portable or stationary instrumentation requires an individual to wear a face mask which is often perceived uncomfortable.

**Fig. 1:**
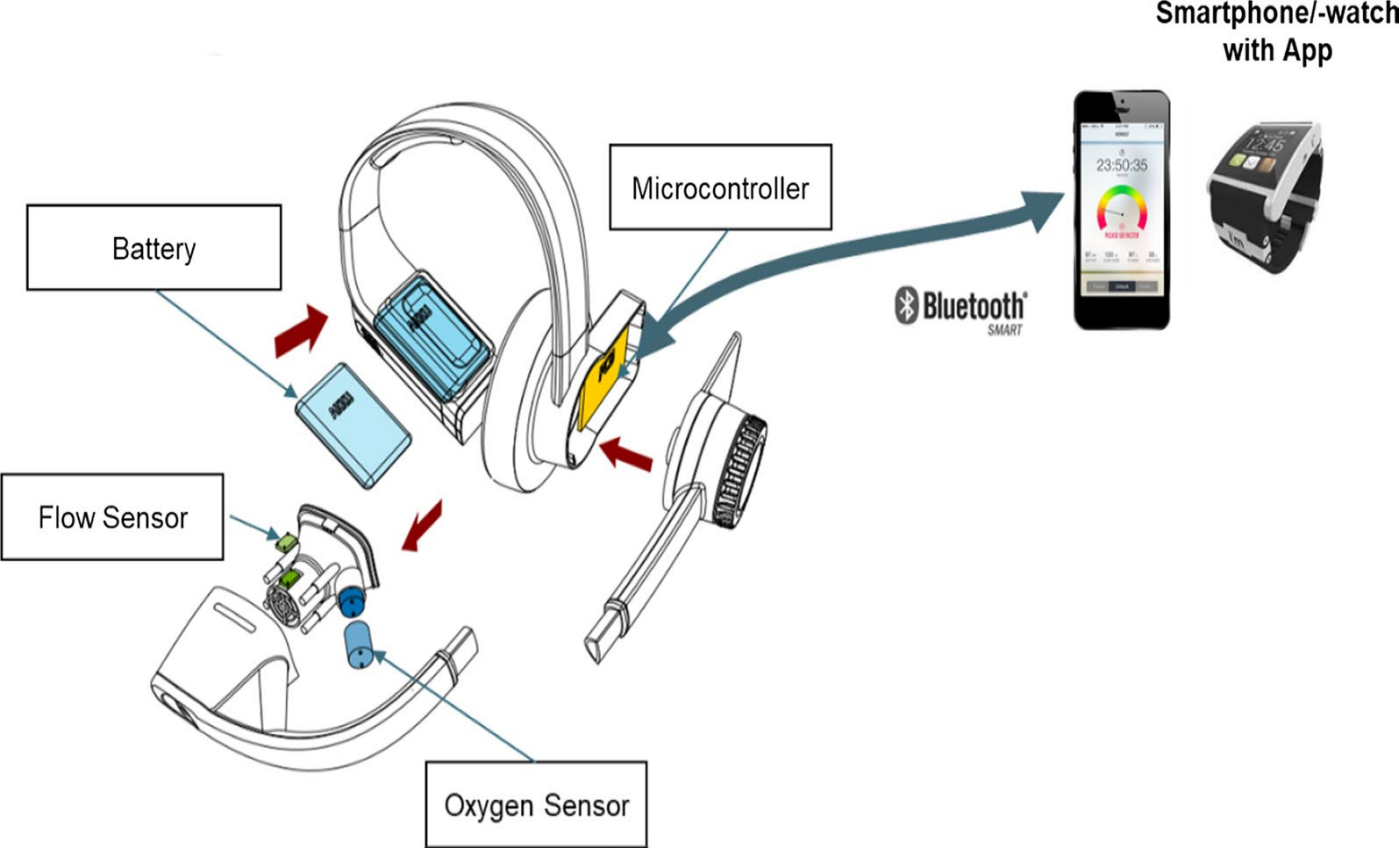
Functional architecture and setup of the prototype headset

To overcome these limitations, a newly developed headset (VitaScale, Nürnberg, Germany) may allow to measure the expired fraction of oxygen and volume flow in the mainstream. The mainstream oxygen and flow sensor alignment allows new form factors within a headset design rendering the use of a face mask unnecessary and making the device smaller and more lightweight than other portable systems (Fig. 1). The price range of the headset is targeted to correspond to the costs of a modern smartwatch which potentially allows the headset to be used by a wider population and for various free-living intraday assessments. For practical purposes and to ensure scientific quality criteria, the data obtained from the headset must be valid, i.e. there must be a high correlation between an established "gold standard" criterion and the new device [7–10]. However, criterion measures also show a certain degree of inaccuracy due to technical errors and within-subject variability. Consequently, it is also important to assess the test–retest reliability error rates of the criterion measure since this magnitude of error has to be taken into account for the between-device (i.e. criterion measure vs new device) validity interpretation.

The aims of the present investigation were twofold: (i) To disclose the technical setup of a headset incorporating a gas and volume sensors allowing to measure expired oxygen concentration and volume flow; (ii) to assess the concurrent criterion-validity of the headset to measure V̇O_2peak_ in 44 individuals exercising on a stationary cycle ergometer in consideration of the test–retest reliability of the criterion-measure.

## Main text

### Materials and methods

#### Participants

44 participants (34 male, 10 female, age 26 ± 7 years, body height 178 ± 8 cm, body mass 77.2 ± 12.7 kg) of Caucasian origin were informed about all experimental procedures and provided written consent to participate. Participants were included only if they were free from any injury and/or illness for at least 6 months, non-smokers and accustomed to performing endurance type sports. The study was approved by the institute’s ethical committee (Number: Ethik/Intex/JMU/2020-01) and performed in accordance with the declaration of Helsinki.

### Experimental procedures

After familiarization with all testing procedures, participants visited the laboratory on two occasions, 3–6 days apart for validating the newly developed headset. Participants were fitted in random order with the criterion measure (Cortex Metamax 3 B, Leipzig, Germany) on one visit and the headset on the other. Instruments were employed as indicated by each manufacturer. Laboratory temperature was controlled at 18.5 ± 0.7 °C. On each visit, participants performed an incremental all-out cycling test followed by a 5-min recovery period and a verification phase on a stationary cycle-ergometer (Cylcus 2, RBM Elektronik-Automation GmbH, Leipzig, Germany). Tested measurement errors of oxygen uptake and ventilation of the headset include the technical error as well as biological within-subject variation. On a third occasion, we tested the reliability (i.e. combined biological within-subject variability as well as technical error) of the criterion measure with a group of 13 individuals.

### Testing protocol

For all assessments we employed a prototype version of the headset. Participants had to wear a nose clip to ensure oral breathing. All participants warmed up at 0.6 W kg^−1^ body mass for 5 min. Afterwards, resistance of the cycle ergometer increased by 20 W every 30 s until full volitional exhaustion. Following a 3-min break, the participants performed a verification phase with an intensity 10% higher than their final stage during incremental cycling. All participants were instructed to maintain a pedaling rate of 90 rpm throughout the test. Peak power output was obtained from the stationary cycle-ergometer. After cessation of the incremental test, capillary blood from the right earlobe was analyzed (Lactate Pro 2, Arkray KDK, Japan) to assess blood lactate levels. State of exhaustion of each participants was verified when three of six criteria were met: (i) a respiratory exchange ratio > 1.1 (for the criterion measure test only); (ii) plateau in oxygen uptake (i.e., elevation of ≤1.0 ml min^−1^ kg^−1^ as the velocity was increased); (iii) heart rate within 5% of the age-predicted peak heart rate; (iv) a capillary blood lactate concentration > 6 mmol L^−1^; (v) self-reported rating of perceived exertion > 18 and (vi) a pedaling rate < 90 rpm. All oxygen uptake data was averaged in 30-s intervals and the highest values defined as V̇O_2peak_.

### Criterion measure

A portable breath-by-breath gas analyzer (Metamax 3B, CORTEX BiophysikGmbH, Germany) for assessing V̇O_2peak_ served as criterion measure. This device is commonly used in exercise science research to assess V̇O_2peak_ [11]. However, all criterion-measure display error rates (stemming from technical error and the within-subject random variation) [12] and therefore we calculated reliability measures for the criterion-measure in our respective sample population and test arrangement.

The criterion measure was calibrated before each test using high precision gas (15.8% O_2_, 5% CO_2_ in N;  CORTEX BiophysikGmbH, Germany) and a 3-L syringe for volume flow calibration. The criterion measure provides reliable data with technical measurement error below 2% [5]

### Description of the prototype headset

The headset (dimensions: 300 × 200 × 150 mm, mass: 150 g) included the following technology: an oxygen and volume flow sensor, a data transmitting unit, and a smartphone companion app (Fig. 1  and Additional file 1). All data was transmitted via Bluetooth low energy to a companion iOS app for direct feedback and data storage.

A differential pressure sensor (SPD3x, Sensirion, Switzerland) detected changes in expired air flow (sampling rate: 2 kHz at 16 bit). The recalibrated sensor incorporated temperature compensation thereby rendering regular calibration unnecessary. Detailed technical specifications of the sensor are summarized in the supplementary files. A moisture-proof solid-state electrolytic sensor chip developed by VitaScale (VitaScale GmbH, Nuremberg, Germany) detected the fraction of oxygen in the breathing air (sampling rate: 60 to 100 Hz). The developed sensor allowed calibration with clean ambient air without the need of precision gases for calibration purposes.

### Statistical analysis

A dependent t-test assessed difference of blood lactate concentration and peak power output data between exercise tests.

Statistical analysis was performed in accordance with previous recommendations [13] and similar studies [14] employing a custom Microsoft Excel spreadsheet [15]. To avoid bias resulting from non-uniformity of error, data were log-transformed prior to analysis. Linear regression was employed to analyze validity [13, 16]. For validity analysis, standardized mean bias, standardized typical error of estimate (sTEE), the coefficient of variance (CV%) and Pearson’s product-moment correlation coefficient (Pearson’s r) were calculated. Blant–Altman plots (including 95% confidence limits) analyzed agreement between the two different measurements of V̇O_2peak_.

For reliability analysis of the criterion measure, the standardized typical error (sTE), the CV% and Pearson’s r are reported. Also, 90% confidence limits for statistical parameters (except Blant-Altman plots) are described.

### Results

Data from 10 participants were excluded from further analysis due to handling errors (e.g. false positioning of the headset during measurement, loss of connectivity to the companion app). No differences in maximal power output (p > 0.92) and maximal blood lactate values (p > 0.35) were detected between tests when applying the criterion measure and headset. In comparison to the criterion measure, the headset showed a standardized mean bias of -0.01 (90% CL− 2.77 to 2.74), a Pearson’s r of 0.95 (90% CL 0.91–0.97), a CV% of 6.8% (90% CL 5.6–8.7) and a sTEE of 0.33 (90% CL 0.24–045) (Table 1).

**Table 1:** Validity of peak oxygen uptake measurement between the criterion measure and the VitaScale and reliability of the criterion measure while cycling at different intensities

Statistical parameter	Peak oxygen uptake (90% confidence limits)	
	Validity between vitascale and criterion measure	Reliability of the criterion measure
Standardized mean bias	− 0.01 (− 2.77–2.74)	Cannot be calculated
Pearson’s r	0.95 (0.91–0.97)	0.96 (0.89–0.99)
Coefficient of variance (%)	6.8 (5.6–8.7)	4.0 (3.0–6.3)
Standardized typical error of estimate	0.33 (0.24–0.45)	Cannot be calculated
Standardized typical error	Cannot be calculated	0.21 (0.16–0.33)

**Fig. 2:**
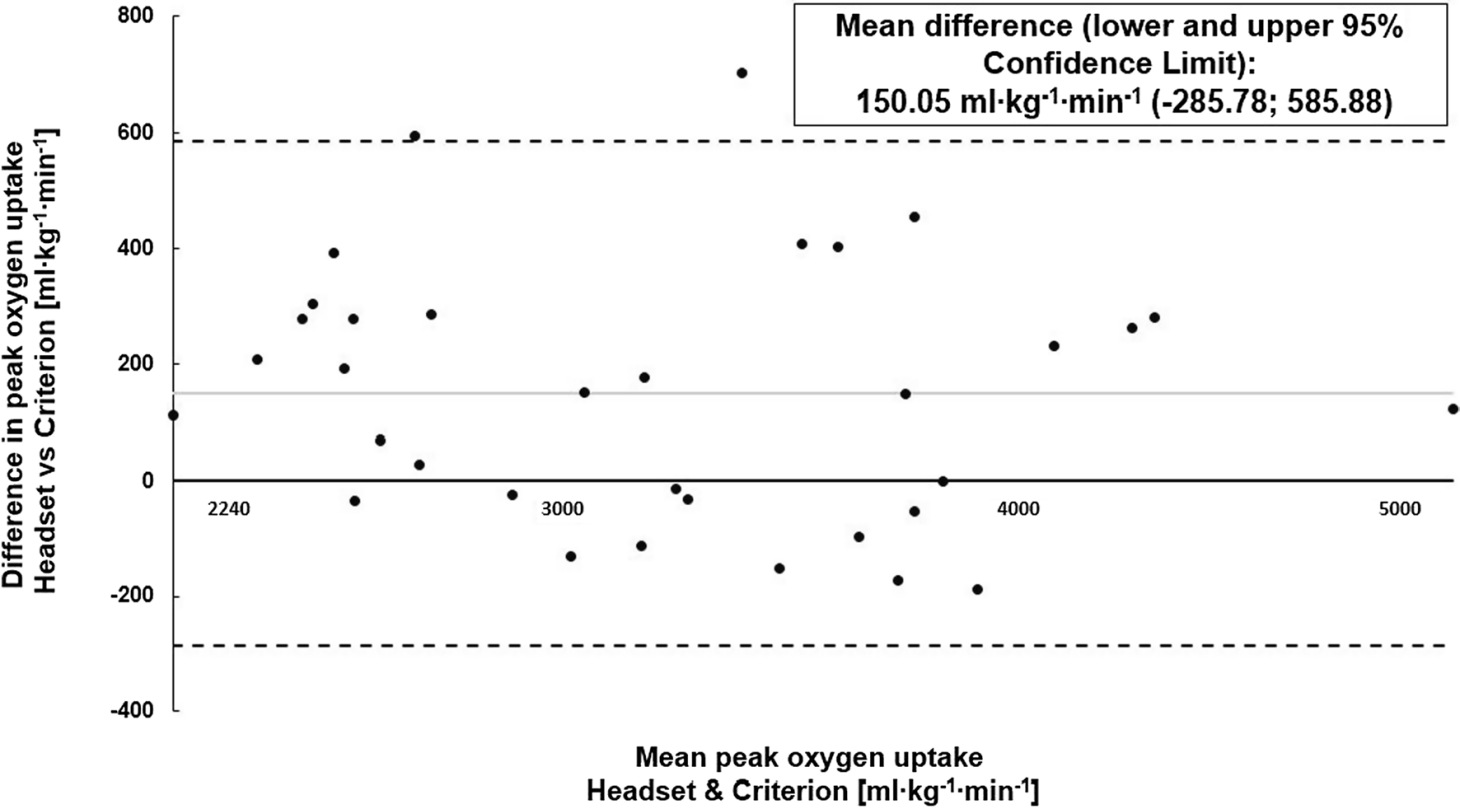
Bland–Altman Plot for peak oxygen uptake between the headset and criterion measure at different intensities including 95% confidence limits

Bland–Altman plots for V̇O_2peak_ are displayed in Fig. 2. Mean difference (lower to upper limits of agreement) between the criterion as well as headset are 150.0 (− 285.7 to 585.8) ml∙min^−1^∙kg^−1^.

Results of the reliability analysis for the criterion measure are displayed in Table 1.

### Discussion

While exercising on a stationary cycle ergometer, the headset (i) measures V̇O_2peak_ with a CV of 6.8% compared to the criterion measure and (ii) in the given population and test arrangement, the criterion measure showed an error (expressed as CV) of 4.0% for V̇O_2peak_. Due to the specific design of the headset and the criterion measure, it is impossible to compare both devices within the same exercise test. Consequently, both devices were tested on two separate occasions thereby increasing the between-test error rate. Although maximal power output and maximal blood lactate values obtained at the end of both incremental tests did not differ (indicated by similar exertion between both test occasions) we cannot preclude that the risk of error which we report here may arise from within-subject variability. However, in the present test arrangement the repeated measure of the criterion device revealed a reliability of 4.0 CV% for V̇O_2peak._

Depending on the study design and exercise modalities, other portable breath-by-breath gas analyzers (employing a fascial mask) show different measurement errors. The MetaMax 3B shows a technical error of < 2% however may overestimate oxygen uptake by 10–17% at moderate and vigorous cycling when compared to a Douglas Bag [5]. The MetaMax3B demonstrated an error of 2.8% for V̇O_2peak_ [17] in individuals repeatedly exercising on an rowing ergometer and an error of 4.1% for oxygen uptake when compared to a Douglas Bag [17].

Since we must accepted an inaccuracy of 4.0 CV% for the criterion measure (in the given test arrangement) and the headset validity was calculated with 6.8 CV% one could argue that practitioners must accept an additional error rate of approximately 2.8% when using the headset. For example, with a “true” V̇O_2peak_ of 60 ml min^−1^·kg^−1^ a participant might display a V̇O_2peak_ of 62.4 ml min^−1^·kg^−1^ when measured with the criterion measure and a V̇O_2peak_ of 64.1 ml min^−1^·kg^−1^ using the new device.

The accuracy of breath-by-breath gas analyzers depends on the responsiveness of the sensor which is defined by the sampling frequency and algorithms converting sensor signals to values such as oxygen uptake [6]. The responsiveness of a gas sensor is described by the time needed to record 10% to 90% of a step change in gas concentration (t90) [6]. According to the manufacturers’ information, the t90 of the headset sensor is 10 ms. Available breath-by-breath breathing gas analyzers indicate a t90 of < 100 ms (Cortex Biophysik GmbH, 2017). Based on the t90 of the headset, the headset O_2_ sensor might detect changes in gas concentration more accurately compared to sensors embedded in available analyzers.

### Conclusions

The newly developed miniaturized headset provides V̇O_2peak_ during incremental cycling with a coefficient of variation of 6.8% compared to a criterion measure in the given research setting. More research is needed employing the headset in different populations, settings and exercise intensities (e.g. submaximal intensity).

### Limitations

(i) 10 sets of tests were discarded due to handling errors (e.g. false positioning of the headset), (ii) the generalizability of the present analysis may be limited for other populations and other settings, (iii) participants wore a nose clip, (iv) submaximal parameters and data from the final design arrangement of the headset should be validated in future studies.

## Supplementary Information

**Additional file 1:** **Figure S1.** Graphical display of the headset and included sensors and technologies (BLE=Bluetooh Low Energy, PCB=Printed Circuit Board,O_2_= Oxygen, CO_2_= Carbondioxide).

## Data Availability

All data are available from the corresponding author on request.

## Abbreviations

CV%: Coefficient of variation
Pearson’s r: Pearson’s product-moment correlation coefficient
sTE: Standardized typical error
sTEE: Standardized typical error of estimate
t90: Time needed to record 10% to 90% of a step change in gas concentration
V̇O_2peak_: Peak oxygen uptake
