# Supplementary material for: Proof-of-concept and concurrent validity of a prototype headset to assess peak oxygen uptake without a face mask
Source: BMC Res Notes. 2022 Jan 8;15:4. doi: 10.1186/s13104-021-05850-y (PMC8742938; doi:10.1186/s13104-021-05850-y)
Supplement: Supplementary file 1 — Additional file 1: Figure S1. Graphical display of the headset and included sensors and technologies (BLE=Bluetooh Low Energy, PCB=Printed Circuit Board,O2= Oxygen, CO2= Carbondioxide). [file 13104_2021_5850_MOESM1_ESM.docx]

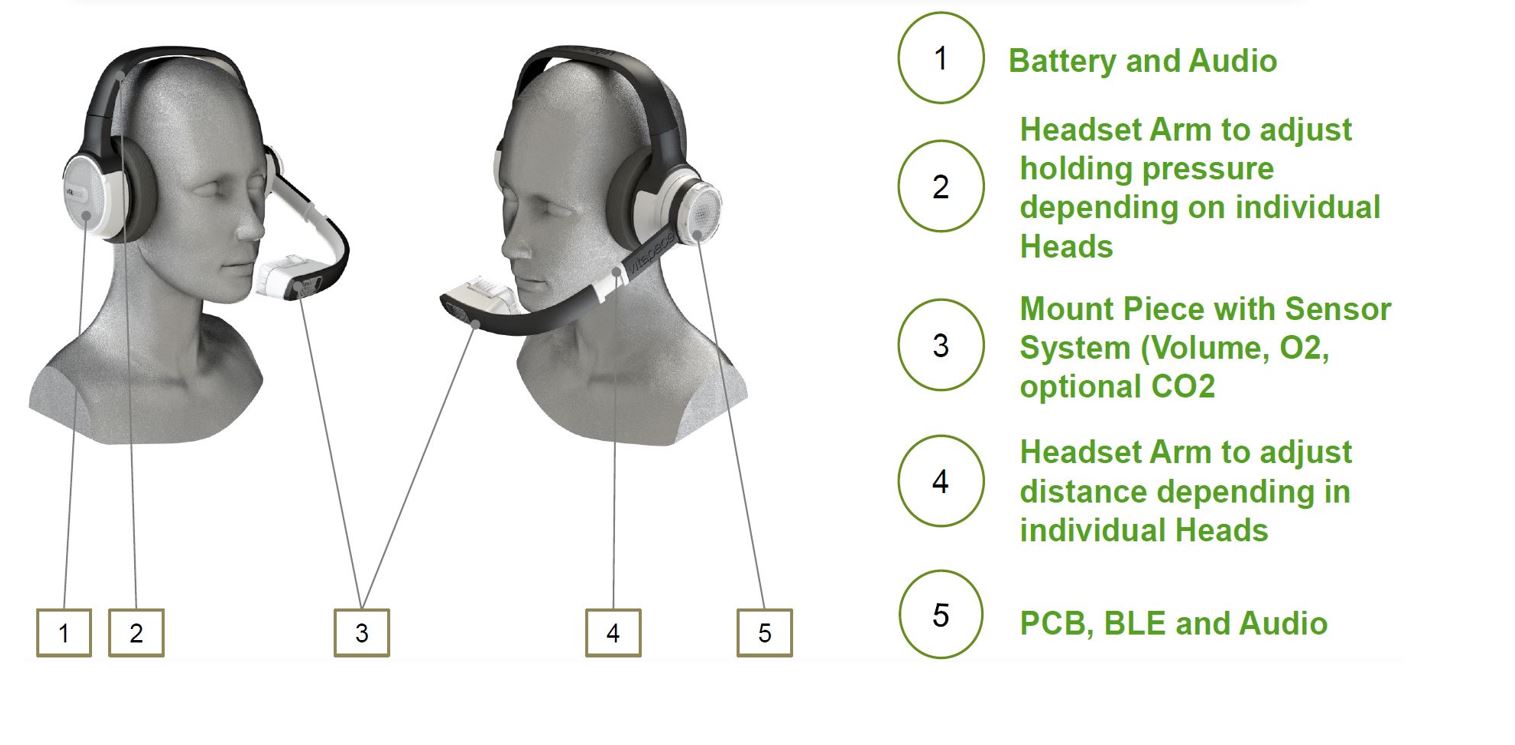


Supplementary figure 1: Graphical display of the headset and included sensors and technologies (BLE=Bluetooh Low Energy, PCB=Printed Circuit Board,O_2_= Oxygen, CO_2_= Carbondioxide)
